# Cases of Hysteritis Puerperalis

**Published:** 1829-09

**Authors:** James Paxton

**Affiliations:** Member of the Royal College of Surgeons in London.


### HYSTERITIS PUERPERALIS.

*Cases of Hysteritis Puerperalis.* By James Paxton, Member of the Royal College of Surgeons in London.

Mrs. B., æt. twenty-eight years, of a florid complexion and sanguineous temperament, had been married ten years, but had never been pregnant till the present year. The usual time of utero-gestation was passed without any of that troublesome sickness, or derangement of health, so often attendant on it. On the 21st of November, 1828, labour commenced: the process went on favorably and naturally, and at the end of four or five hours Mrs. B. gave birth to a well-formed female child. The placenta was expelled, with very little assistance, in a quarter of an hour afterwards. Quietness and composure were enjoined; but the injunction was not complied with: for the gratification of having become a mother after the lapse of an almost hopeless term of years, appeared to give rise to an excitation too powerful for the due exercise of the vital functions. In half an hour, distressing symptoms of hysteria came on, as screaming, sense of suffocation, pale cadaverous countenance, clammy sweats, and coldness of the extremities. On examination, there was found considerable uterine discharge, and a great sense of exhaustion followed.—Wine and water was given, and Ammonia cum Tr. Opio ♏xl.

22d.—The patient had a quiet night; but this morning she complains of pain in the loins and tenderness about the hypogastric region. Passes water frequently, but in small quantities, and there is a very considerable lochial discharge. Pulse 125; tongue dry; thirst; surface hot and dry, and looks pallid.—Calomel gr. viij. statim, et Haust. Salin. quartis horis.

23d.—Had copious alvine evacuations; less pain, some sleep; pulse 120.—Pergat in usu medicamen. præscript. sine Calomelane.

24th.—Complains of headach; has had chills, alternating with flushings of heat; acute and permanent pain in the same situation as above described. When pressure is made, there is extreme tenderness of the abdomen, but no tension; there are also wandering pains over the whole body. Uterine hemorrhage continues.

25th.—Haust. Inf. Rosæ cum Magn. Sulph. ʒi. quartis horis.—There is some abatement of the former symptoms. Large coagula were expelled from the vagina during the night.—Haust. Infus. Rosæ cum Acid. Sulph. dil. quartis horis. Ol. Ricini ʒvi. statim.

28th.—Up to this period no material change has occurred. The pulse is small and frequent; tongue covered with a light brown fur on the back of it; temperature of the skin beyond the natural standard; perpetual desire to void the urine, which is scanty, deep coloured, and turbid. Lochial discharge continues in considerable quantity. No milk has been secreted. Complexion sallow. On some occasion the patient was removed from her bed, when syncope and clammy perspiration alarmed her attendants. She has no appetite, but is thirsty. There is some degree of fulness and uneasiness in the abdomen, but not amounting to pain, unless pressure is made with the hand, which discovers the uterus to be thrice its unimpregnated size.—Emp. Canth. supra regionem hypogast. applicandum. Pergat in usu medicamen.

December 3d.—Some relief was procured: the pains were very much diminished; and, on the whole, it may be said that the train of unpleasant symptoms and constitutional disturbance is subsiding. Pulse 105. Sometimes small doses of Ammon. Subcarb. was exhibited; at others, effervescing medicine and occasional doses of Ol. Ricini.

This state of things continued, with little variation, till the 8th, when marked rigors, sweats, and diarrhœa, suddenly, and certainly unexpectedly, supervened. Wine and opiate confection were administered; but the following morning (the 9th,) respiration became laborious, the sight dim, mind wandering, pulse scarcely perceptible; profuse colliquative perspirations bedewed the body; and in the night the patient expired.

*Sectio cadaveris.*—The body was examined about twelve hours afterwards, in the presence of Dr. Kidd. The liver, stomach, and intestines, exhibited no morbid affection of any kind. There was no effusion or unhealthy appearance of the peritoneum. The disease was found to be limited to the uterus: this organ was five inches in diameter; its peritoneal covering had some slight pencilling of vascularity; but its internal structure had undergone very extensive change. The whole inner surface was of a dark crimson and livid hue; the cervix was completely gangrenous, and gave forth a highly offensive vapour.

The second case occurred on the 24th of May, 1829, on which day Mrs. H. fell in labour with her second child. A midwife attended her for fourteen hours; the membranes were ruptured, and very considerable hemorrhage took place, producing great faintness. A medical gentleman (Mr. Tomes) was therefore called in, who, very properly, immediately adopted an opposite plan to that which had been hitherto pursued. Instead of warm stimulants, with which she had been plentifully supplied, he ordered cold liquids, and sulphuric acid with infusion of roses. This succeeded in suppressing the hemorrhage. The pains were trifling. On examination, the hand of the child was found to have fallen into the vagina. Mr. Tomes then requested my attendance. I advised the extremity to be replaced, and the child to be turned; but, from the rigidity of the uterus, its powerful contraction, and from the large size of the child, there was a delay of several hours before this object could be accomplished. The feet were at length brought down, and the body and head were then delivered without difficulty. The placenta was not long detained. A sense of excessive fatigue and faintness immediately succeeded, and a recurrence of hemorrhage.—Took Tr. Opii ♏xl.

25th.—The patient was very restless, sighing, and experienced great pain in the back and hypogastric region. Pulse 140.—Calomel gr. x.; Opii gr. ij. statim. Haust. effervescens quartis horis.

26th.—More comfortable; pulse 110. But in the evening, chills and cold perspirations were frequent, and the pain increased.—Calomel gr. viij.; Opii gr. ij. statim. Pergat in usu haust.

27th.—The patient feels less pain, and has had some sleep.—Pergat.

28th.—Passed a restless night, and the pain has returned with greater violence. There is a sense of exhaustion, fainting, anxiety, and general uneasiness: in particular, pain over the uterus, which was increased on the most moderate pressure; but there is no abdominal tension. Lochial discharge in considerable quantity.—Applicentur Hirudines xx.

Somewhat relieved; but the circumscribed swelling and tenderness at the lower part of the abdomen continue, and in the evening complained of great pain and tenderness about the os uteri, rigors or heats, depression of spirits, and general uneasiness.—V.S. ad ℥xxiv. Haust. efferves. cum Ant. Tart. gr. $\frac{1}{4}$ tertiis horis.

The blood drawn exhibited marks of inflammation. The pain and sensibility, however, was much diminished, and from this time there may be stated to have been a rapid amendment, until the 6th of June, when a slight attack of phlegmasia dolens supervened, and protracted the cure for three weeks longer; since which the patient has been free from complaints, and, indeed, about her domestic occupations.

The reflections I make on a comparison of these cases are,

1st. That copious depletion is the most powerful means of subduing inflammatory action of the uterus.

2d. That uterine discharges have no effect in relieving that organ, when suffering under inflammation.

3d. That neither the faintness experienced by the patient, nor even uterine hemorrhage, or weakness of the pulse, should have any weight on the mind of the practitioner, so as to prevent his carrying local or general bloodletting to its requisite extent: for if there is fever, with constant uterine and general pain, this is the true criterion for forming a judgment of the propriety of the measure, and not any other consideration whatever.

*Oxford; July* 16*th*, 1829.

